# Telemedicine Acceptance Among Older Adult Patients With Cancer: Scoping Review

**DOI:** 10.2196/28724

**Published:** 2022-03-29

**Authors:** Ning-Qi Pang, Jerrald Lau, Si-Ying Fong, Celine Yu-Hui Wong, Ker-Kan Tan

**Affiliations:** 1 Division of Hepatobiliary & Pancreatic Surgery and Liver Transplantation National University of Singapore Singapore Singapore; 2 Yong Loo Lin School of Medicine National University of Singapore Singapore Singapore; 3 Saw Swee Hock School of Public Health National University of Singapore Singapore Singapore; 4 Division of Colorectal Surgery University Surgical Cluster National University Hospital Singapore Singapore

**Keywords:** older adult patients, cancer, telemedicine, acceptability, satisfaction

## Abstract

**Background:**

Cancer is likely to remain the most prevalent noncommunicable disease in high-income countries with an older population. Interestingly, no review of attitudes toward telemedicine among older adults has been performed. This is likely to be the group most affected by both cancer and the increasing use of technology in health care.

**Objective:**

We aimed to map research on the acceptance of telemedicine among older adults who are cancer patients.

**Methods:**

We conducted a scoping review. PubMed, EMBASE, PsycINFO, CINAHL, and the Cochrane Central Register of Controlled Trials were systematically searched from inception to September 2020. Articles were included if the study population had a mean or median age ≥65 years, with cancer diagnoses and if the study assessed patients’ acceptance of a telemedicine intervention. Quantitative, qualitative, and mixed method studies were included.

**Results:**

Out of a total of 887 articles that were identified, 19 were included in the review. Interventions were delivered via telephone, videoconference, web portal, mobile app, wearable technology, and text messaging and included teleconsultation, monitoring and follow-up, psychosocial support and nursing care, and prompts. The most often cited facilitating factor was convenience. Other facilitators included an increase in telemedicine care accessibility, previous positive experiences of telemedicine, appropriate technical knowledge and support, decreased cost, physician recommendations, and privacy conferred by the telemedicine intervention. Barriers include a preference for conventional care along with negative perceptions of telemedicine, concerns about technical difficulties, and confidentiality concerns in the adoption of telemedicine.

**Conclusions:**

None of the studies explored the ability of tailored interventions to address facilitators and barriers of the acceptance of telemedicine in order to increase its adoption by older adults. Facilitators and barriers will likely differ across different cultural contexts and by type of telemedicine; however, this is a gap in current knowledge. In-depth studies are necessary to determine if interventions could potentially address the barriers identified in this review, to increase acceptability.

## Introduction

Cancer is the among the most prevalent of noncommunicable diseases in high-income countries [1], and the general incidence is likely to rise with a greying population. Correspondingly, the number of older adults who are cancer patients requiring cancer care will only continue to increase [2]. That said, cancer care in the current era is increasing in complexity, with newer and better treatment options in aspects of care such as surgery, nutritional needs, nonsurgical treatment, wound care, and cancer surveillance [3]. Despite the amazing speed of technological adoption across different aspects of health care which are purportedly patient-centric (ie, bringing the service to the patients), care for patients with cancer remains very hospital-centric—the number of required visits to the hospital remains considerable for every patient. One mode of reducing hospital visits is the adoption of telemedicine, which has been accelerated by the COVID-19 pandemic [4-6].

Interestingly, there is no universal definition of *telemedicine*—one study [7] established 104 peer-reviewed definitions of the term. The World Health Organization defines *telemedicine* as “the delivery of health care services, where distance is a critical factor, by all health care professionals using information and communication technologies for the exchange of valid information for diagnosis, treatment and prevention of disease and injuries, research and evaluation, and for the continuing education of health care providers, all in the interests of advancing the health of individuals and their communities [8].” Based on this definition, telemedicine can take many forms, ranging from a simple phone call to televideo consultations and other potential applications.

While telemedicine could represent the next paradigm shift in global models of health care, barriers to its widespread adoption are present and can range from system-wide logistical issues to individual-level resistance, affecting patients, health care providers, and administrators. Crucially, while telemedicine adoption and use might be more intuitive in younger working adults who are likely to be more technologically savvy, it remains debatable how the incorporation of telemedicine into models of health care would be perceived among older adult patients, especially those with critical illness such as cancer [9].

The rapidly rising incidence of cancer among older adults [10] and the high demands of oncology care mean that this group of patients could potentially benefit immensely from the adoption of telemedicine; yet, there is also a risk that, instead, this population will be left behind by advances in technology. Telemedicine adoption is complex, and system- or community-level frameworks to explain socioenvironmental drivers—such as health economic or diffusion of innovation models—exist [11]. However, individual behavioral factors continue to play an important role in predicting telemedicine adoption at the inter- and intrapersonal level [11]. In this context, technology acceptance has often been identified as an important precursor to adoption of telemedicine initiatives. A comprehensive review [12] illustrated the applicability of technology acceptance models with respect to telemedicine acceptance; one of the most frequently utilized [12,13] theoretical models identified was the Unified Theory of Acceptance and Use of Technology (UTAUT) [11]. While *acceptance* is not defined within the UTAUT model, the concept broadly involves an individual’s behavioral use of the technology or system [13].

In the UTAUT model, performance expectancy, effort expectancy, social influence, and facilitating conditions are 4 constructs that are direct determinants of behavioral intentions, and thus, of behavioral use: *performance expectancy* is defined as the degree to which an individual believes that using the technology will help in completing the task; *effort expectancy* is defined as the degree of ease of use of the technology; *social influence* is defined as the individual’s perception of whether important people around them believe that the individual should use the technology; and *facilitating conditions* is defined as the degree to which an individual believes that technical expertise and infrastructure are available to support the use of the technology [13].

Despite widespread use of *acceptance* as a concept to explain telemedicine adoption in the context of chronic diseases (eg, hypertension) and population (eg, caregivers, older adults), reviews, specifically on telemedicine for older adults who are patients with cancer [14-16], have not addressed telemedicine acceptance. Thus, we aimed to systematically map the international body of literature on acceptance of telemedicine among older adults who are patients with cancer and frame our current understanding of this topic within the UTAUT model.

## Methods

### Review Protocol and Search Strategy

#### Protocol

We based our review methodology and structure on an established example [17]. A protocol was developed *a priori,* and registered on PROSPERO (CRD42021235248); we elected to utilize PRISMA-ScR (Preferred Reporting Items for Systematic Reviews and Meta-analysis for Scoping Reviews [17]) as the framework because the objective was to map existing evidence on telemedicine acceptance among older adult cancer patients, to identify the major knowledge gaps. The research question was defined using the PICOS (population, intervention, comparison, outcome, study design) framework. Specifically, we were interested in all quantitative, qualitative, or mixed methods studies (both observational and interventional studies) (study design) that examined older adult cancer patients’ (population) acceptance (outcome) of telemedicine initiatives (intervention). Where possible (eg, if a study utilized an interventional design), we were interested in telemedicine compared with standard (eg, face-to-face) care (comparison) (Table S1 in Multimedia Appendix 1).

#### Research Question

What is known from the literature about older adults (defined as ≥65 years old) who are cancer patients and their acceptance of the use of telemedicine?

#### Search Strategy

Based on the protocol, 2 reviewers (SYF, CYHW) conducted a comprehensive search of PubMed, EMBASE, PsycINFO, CINAHL, and the Cochrane Central Register of Controlled Trials. PubMed, EMBASE, and CINAHL were selected based on recommendations [18] on best databases for reviewing telemedicine-related topics. PsycINFO and the Cochrane Central Register of Controlled Trials were used because of the possibility that relevant studies examining telemedicine acceptance might have been published in behavioral psychology journals or as trials, respectively. Peer-reviewed papers were included if they were published before September 2020 and written in English. The concepts searched were relevant to telemedicine (eg, telemedicine, telehealth), attitudes toward telemedicine, and cancer patients (eg, neoplasm). Search terms were broad (for example, the Medical Subject Heading *attitude to health*) and applied to all fields (rather than simply to titles, abstracts, and article keywords) to minimize the likelihood of excluding relevant articles. The search strategy was constructed using free-text keywords and Boolean operators. The search strategy was refined through discussions among all coauthors (Table S2 in Multimedia Appendix 1).

To further reduce the likelihood of missing relevant articles, we manually searched the reference lists of articles included in the review.

### Inclusion and Exclusion Criteria

First, as our study population of interest was older adults (defined as 65 years or older), we included only articles that had a study population with an average (mean or median) age that was 65 years or older. This ensured that there was a majority representation (at least 50% or more) of views of older adults in the reported outcomes. Studies that did not report mean or median age were, therefore, excluded. Second, only studies involving patients with cancer were included; cancer was defined as a malignant growth or tumor resulting from an uncontrolled division of cells. Third, studies must have included the use of telemedicine. Given that there has been no clear definition of telemedicine in the literature [7], in this review, any form of technology utilization to aid in delivery of health care was considered to be telemedicine. Fourth, patient acceptance of the telemedicine studied must have been reported. Acceptance was defined as an individual’s likely behavioral use of the technology or system [13]. Participants may or may not have actually used the technology studied, as long as their perspective on the technology had been reported. Only studies reporting patient perspectives were included. Studies reporting perspectives of health care providers or caregivers were excluded. Grey literature (eg, news articles, lecture slides, unpublished theses), reviews, editorials, letters to the editor, and studies not published in English or in peer-reviewed journals were excluded.

### Selection of Articles

Duplicates were removed from the list of articles returned by the searches (manually and using EndNote X8). Two reviewers (SYF, CYHW) screened the titles and abstracts of all the articles independently to select articles for full-text review. Articles were included if at least one reviewer deemed it potentially suitable. Four reviewers (NQP, SYF, CYHW, JL) were involved in the review of full-text articles. Each full text was independently reviewed by at least 2 of the reviewers: full texts were evenly distributed among reviewers, and a second reviewer was assigned to independently verify each set, with moderate (based on [19]) levels of agreement overall (SYF and NQP: Cohen κ=0.58; CYHW and SYF: Cohen κ=0.49; JL and CYHW: Cohen κ=0.46; NQP and JL: Cohen κ=0.86). Disagreements were resolved by discussion, among all 4 reviewers, until consensus was reached.

### Data Extraction and Analysis

Data, relevant information on key study characteristics, and detailed information on the acceptance of telemedicine among older adults patients with cancer were extracted from the articles included in the review. Two reviewers (SYF, CYHW) independently charted data, discussed the results, and iteratively updated the data-charting form. The final data fields collected included the aim of the study, background or context, average (mean or median) age of participants, type of telehealth intervention (eg, teleconsultation, monitoring, nursing), telehealth technology used, presence of comparator arm, duration of intervention, outcome measured, methods of measuring outcomes (instrument or scales used), and key findings. Any disagreements were resolved by discussion or adjudication (by a third reviewer, JL).

## Results

### General

**Figure 1:**
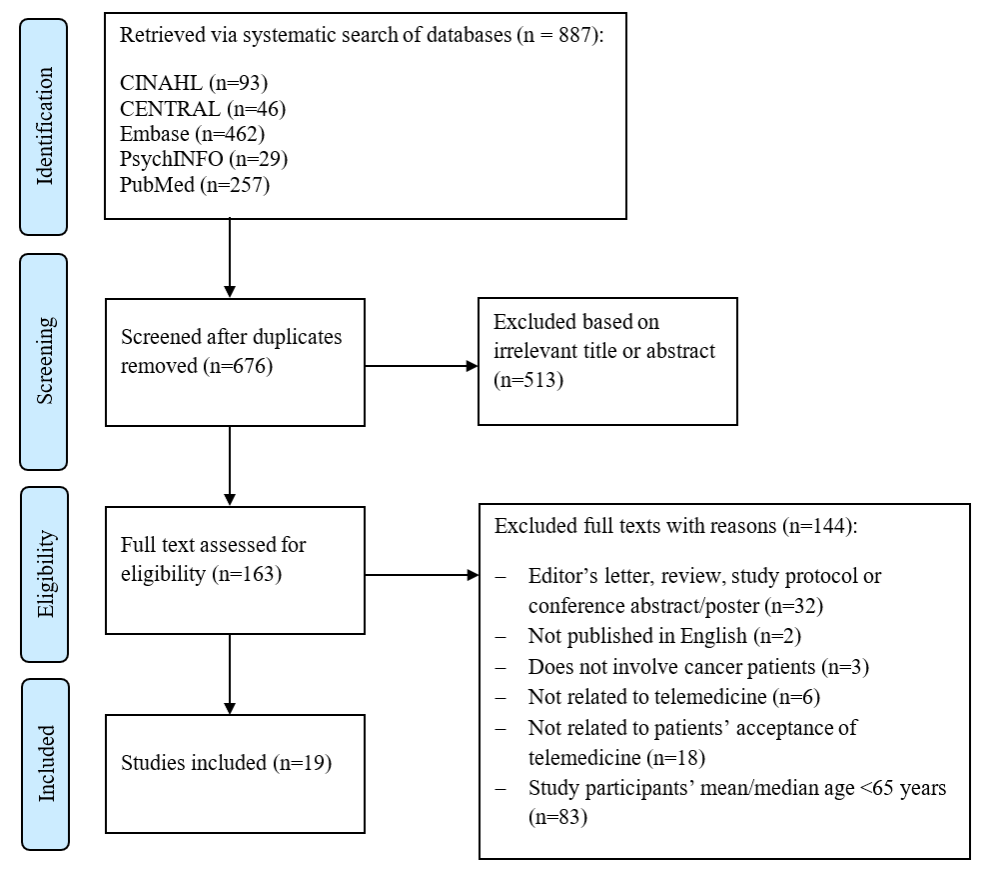
Study selection flowchart.

A total of 887 articles were identified, from which 211 duplicates were removed, leaving 676 articles for title and abstract screening. Of these, 513 articles were excluded, and 163 articles underwent full-text review, during which, 137 articles were excluded (Figure 1; Multimedia Appendix 1)

### Descriptive Characteristics

More than two-thirds (14/19, 73.7%) of studies were performed in the United Kingdom and United States, and the rest were from other high-income countries; none were conducted in low- or middle-income nations. A clear increase in publication frequency was noted after the year 2010, with 21.1% (4/19) of studies published in the period from 2011 to 2015, and 57.9% (11//19) of studies published in the period from 2016 to 2020. More than half (10/19, 52.6%) of the studies were cross-sectional (nonrandomized trial: 2/19, 10.5%; pre- and posttest: 1/19, 5.3%; randomized controlled trial: 2/19, 10.5%: qualitative: 4/19, 21.1%). Sample sizes ranged from n≤50 (6/19, 31.6%) to n>300 (1/19, 5.3%). The 19 studies included a range of different cancers (Table 1).

**Table 1:** Population and study characteristics.

Characteristics			Articles (n=19), n (%)
**Country**			
	Canada	1 (5.3)	
	Denmark	1 (5.3)	
	Germany	2 (10.5)	
	The Netherlands	1 (5.3)	
	United Kingdom	7 (36.8)	
	United States	7 (36.8)	
**Publication year**			
	1995-2000	2 (10.5)	
	2001-2005	1 (5.3)	
	2006-2010	1 (5.3)	
	2011-2015	4 (21.1)	
	2016-2020	11 (57.9)	
**Study design**			
	Cross-sectional	10 (52.6)	
	Nonrandomized trial	2 (10.5)	
	Pre- and posttest	1 (5.3)	
	Randomized controlled trial	2 (10.5)	
	Qualitative	4 (21.1)	
**Sample size**			
	≤50	6 (31.6)	
	51-100	4 (21.1)	
	101-200	6 (31.6)	
	201-300	2 (10.5)	
	>300	1 (5.3)	
**Site of cancer**			
	Breast	1 (5.3)	
	Colorectal	1 (5.3)	
	Endometrial	3 (15.8)	
	Esophagogastric	1 (5.3)	
	Hematological	1 (5.3)	
	Lung	1 (5.3)	
	Prostate	2 (10.5)	
	Skin	1 (5.3)	
	Mixed	8 (42.1)	

### Range of Technology

Studies included interventions delivered via telephone (6/19, 31.6%), videoconference (4/19, 21.1%), web portal (2/19, 10.5%), mobile app (3/19, 15.8%), wearable technology (1/19, 5.3%), or text message (1/19, 5.3%). Of the 19 articles, 2 articles (10.5%) surveyed patients on technology in general (ie, without specifying a technology). Telemedicine interventions included teleconsultation (2/19, 10.5%), monitoring and follow-up (7/19, 36.8%), psychosocial support and nursing care (5/19, 26.3%), and prompts (eg, to increase treatment adherence) (2/19, 10.5%). Of the 19 articles, 3 articles (15.8%) did not specify a particular telemedicine intervention, and participants in 5 studies (26.3%) did not actually use the technology in question—they were surveyed on their acceptance of that particular technology based on their impression of its usage (Table 2).

**Table 2:** Technology and intervention characteristics.

Characteristic			Articles (n=19), n (%)
**Technology type**			
	Telephone	6 (31.6)	
	Mobile text	1 (5.3)	
	Videoconferencing	4 (21.1)	
	Web portal	2 (10.5)	
	Mobile app	3 (15.8)	
	Wearable technology	1 (5.3)	
	Technology in general	2 (10.5)	
**Type of telemedicine intervention**			
	Teleconsultation	2 (10.5)	
	Monitoring and follow-up	7 (36.8)	
	Psychosocial support and nursing care	5 (26.3)	
	Prompts	2 (10.5)	
	Not specified or general	3 (15.8)	
**Actual usage of technology studied**			
	Yes	14 (73.7)	
	No	5 (26.3)	

### Facilitators and Barriers to Technology Acceptance

Four studies [20-23] used previously validated questionnaires. None used an evaluation tool based on a theoretical behavioral framework. Study topics were categorized into themes (Table 3).

Of the 19 articles, 15 articles reported either facilitators or barriers to explain their findings on acceptance; the other 4 articles did not [20,21,24-26,28-37] (Table 4).

**Table 3:** Key telemedicine acceptance findings.

Type of telemedicine and reference			Type of study		Aim of study		Key findings
**Teleconsultation**							
	Allen et al (1995) [25]	Pre- and posttest study (n=21)		To assess levels of satisfaction (acceptance) among rural cancer patients being seen for clinic visits by using interactive videoconferencing		Patients were less inclined to want to use video system again when asked after attending an on-site consultation (P=.016)	
	Mair et al (2000) [31]	Qualitative (n=22)		To understand patients' views of telemedicine consultations		Participants felt that there was a difference in telemedicine consultations vs face-to-face visits (eg, modified behavior of patients) but were accepting of these differences to increase their access to health care and medical expertise	
**Monitoring and follow-up**							
	Overend et al (2008) [32]	Cross-sectional (n=53)		To determine whether nurse-led telephone clinic could effectively and safely be used to follow patients with indolent and chronic hematological malignancies		62% of participants felt strongly that they would participate in a Teleclinic again rather than travel to see their oncologist	
	Beaver et al (2011) [20]	Cross-sectional (n=187)		To explore patient satisfaction on different aspects of follow-up service provision following treatment for colorectal cancer and amenability to an alternative strategy for follow-up care		66% of patients mentioned that they would be willing to receive telephone follow-up care in the future Male patients were 2 times more likely to indicate willingness for telephone follow-up	
	Verma et al (2015) [27]	Cross-sectional (n=134)		To evaluate a radiographer-led telephone follow-up for patients with low to intermediate risk prostate cancer patients completing radiotherapy		67 out of 88 (76%) expressed a preference for telephone follow-up, while 7% expressed no preference between clinic or telephone follow-up and 5.5% expressed preference for outpatient clinic follow-up	
	Onuma et al (2019) [38]	Cross-sectional (n=271)		To determine patient preferences around the preferred means of receiving information about cancer surveillance (secure digital communication versus phone call or office visit)		Patients >65 years preferred telephone or in-person communication of normal imaging results (ORa 2.03, 95% CI 1.16-3.56, P<.05) versus patients ≤65 years; all patients preferred telephone or in-person consult for abnormal results	
	Smits et al (2015) [22]	Nonrandomized trial (n=296)		To evaluate the effect of nurse led follow-up on quality of life and patient satisfaction compared to conventional follow-up in women treated for endometrial cancer and to evaluate the patient acceptance of nurse-led follow-up		Majority of women (98%) in the nurse-led follow-up group stated that they would like to continue their follow-up care with the nurse-led telephone clinic Women in both groups reported equal satisfaction with care	
	Wynter-Blyth et al (2017) [30]	Pre- and posttest (n=9)		To explore the perioperative potential of home remote-monitoring (eg, on adherence to prehabilitation and rehabilitation programs)		All 9 patients mentioned that they would recommend home remote-monitoring to other patients and 8 out of 9 said that they would consider buying their own personal health monitoring devices Helped to build their confidence in managing their condition and allowed them to play a more active role in their overall health, such as improving their adherence to exercise and diet	
	Nugteren et al (2017) [26]	Qualitative (n=20)		To investigate patients' opinions about the use of eHealth apps to support self-management in survivorship care		Majority of participants would like to use the app as they have positive attitudes toward the app	
**Psychosocial support and nursing care**							
	Xu et al (2014) [21]	Cross-sectional (n=230)		To model intention of lung cancer patients to using face-to-face and online lung cancer support groups		Positive intentions to join an online support group were reported by 34% of participants, whereas for the face-to-face support group, positive intentions to join were reported by 36.4% of participants	
	Beaver et al (2020) [34]	Cross-sectional (n=211)		To explore the preferences of endometrial cancer patients and their levels of satisfaction with hospital vs nurse-led telephone follow-up		Participants tended to prefer what was familiar to them; those in the hospital follow-up group tended to prefer hospital-based appointments while the telephone follow-up group tended to prefer appointments with a clinical nurse specialist, regardless of locality	
	Bohnenkamp et al (2004) [28]	Nonrandomized trial (n=28)		To measure the impact of telenursing on patients discharged with ostomies resulting from cancer treatment (telenursing + home health visit vs only home health visit)		87% said they would prefer telenursing visit over waiting for a face-to-face visit 70% said that they would prefer a face-to-face visit (if no waiting time required) even though 85% agreed that the telenursing visit was as good as a face-to-face visit 93% of patients were satisfied with the telenursing combined with home health visit, while 81% were satisfied with just the home health visit (P<.01)	
	Bouchard et al (2019) [33]	Randomized controlled trial (n=192)		To examine the acceptability and efficacy for reducing disease-specific distress of a tablet-delivered psychosocial intervention for older men with advanced prostate cancer		Average exit survey responses were favorable and similar for intervention (mean 3.53, SD 0.55) and control (mean 3.65, SD 0.41; P>.05)	
	Williamson et al (2018) [35]	Qualitative (n=25)		To explore the views of women with endometrial cancer who had received telephone follow-up compared to hospital follow-up		Patients generally preferred telephone follow-up compared to hospital follow-up	
**Prompts**							
	Spoelstra et al (2016) [24]	Randomized controlled trial (n=75)		To conduct a preliminary evaluation of the efficacy of telemedicine with respect to adherence and symptom severity and interference in adult cancer patients prescribed Oral Anticancer medication		97.4% recommended it as a way to assist patients to remember to take medications and 100% would recommend it to their oncologist as a way to monitor adherence 85.7% of participants completed the entire telemedicine intervention, suggesting that there is high acceptability Majority of participants (92.2%) reported high satisfaction	
	Brett et al (2018) [36]	Qualitative (n=18)		To assess the likely acceptability of an eHealth app in women who have utilized the app to support women prescribed adjuvant endocrine therapy after treatment for breast cancer		All participants except one said that they would recommend the app to women taking adjuvant endocrine therapy	
**Not specified or general**							
	Steeb et al (2019) [37]	Cross-sectional (n=200)		To investigate patient attitudes and their awareness toward skin cancer–related apps		Most patients (86/126, 68.3%) rated scientifically reliable information as the most important feature for health-related apps, followed by user convenience (76/126, 60.3%) and data security (76/126, 60.3%) For 54.0% (68/126) of patients, credibility of the app provider was important 29.6% (37/125) and 25.4% (32/126) considered a low price and an attractive layout as critical, respectively	
	Rossen et al (2019) [23]	Cross-sectional (n=305)		To get insight of how receptive cancer survivors are toward using health technology for physical activity rehabilitation		88 participants (28.9%) were unreceptive toward supplementing their rehabilitation with technology devices	
	Rodler et al (2020) [29]	Cross-sectional (n=92)		To determine patients’ perspective on adoption of telehealth as a response to the COVID-19 pandemic		General sustainability of telehealth beyond pandemic: majority (65.9%) not inclined to continue telehealth measures Type of treatment plays a role in telehealth acceptance: patients on immunotherapy are more willing to continue with telehealth measures than patients on chemotherapy	

^a^OR: odds ratio.

**Table 4:** Facilitators and barriers to telemedicine acceptance.

Reference	Facilitators	Barriers
Allen et al (1995) [25]	N/A^a^	Patients found that it was more difficult to be completely candid over the video consult than during the in-person consultation, when asked after the on-site consultation (P=.024)
Mair et al (2000) [31]	100% of patients expressed positive attitudes regarding satisfaction with telemedicine; mainly due to convenience of access	50% expressed confidentiality concerns, and 50% felt telemedicine cannot fully replace face-to-face consults 41% were uneasy with the nurse as proxy for physical exam
Overend et al (2008) [32]	78% of participants felt strongly that the teleclinic was convenient and/or saved them time and money	Younger patients who lived one to two hours away from the cancer center declined participation in the teleclinic, as they did not consider the distance an inconvenience Some patients took the opportunity to shop when they came for follow-up visits and did not regard it always as an inconvenience
Beaver et al (2011) [20]	Greater satisfaction with the time given by professionals, practical advice, dietary information and comfort in contacting a colorectal nurse between appointments predicted for acceptance of telephone follow-up	N/A
Verma et al (2015) [27]	79 out of 88 patients (92%) who completed the satisfaction questionnaires reported that telephone follow-up was more or equally convenient as compared to clinic attendance	N/A
Onuma et al (2019) [38]	N/A	N/A
Smits et al (2015) [22]	N/A	N/A
Wynter-Blyth et al (2017) [30]	Usability of the equipment was high, with 8 out of 9 participants finding the app and home remote-monitoring devices clear to navigate and easy to use	5 out of 9 patients experienced difficulties in reliability of the equipment, such as connection issues
Nugteren et al (2017) [26]	Perception that the app increases awareness of symptoms, concerns and supportive care possibilities and improves accessibility	Concerns include: use of the app might be dependent on the current mood and state of the patient (eg, if unwell), preference for face-to-face contact, ease of use, need for concise and simple information, personalized advice as well as the importance of privacy
Xu et al (2014) [21]	Greater computer familiarity increases intention to join online support groups	Having more negative attitudes about online support groups decreases intention to join online support groups. Older age, being male and lower levels of education were associated with more negative attitudes about online support groups
Beaver et al (2020) [34]	telephone follow-up group more likely to indicate that follow-up appointments were always on time and more thorough compared to hospital follow-up group. Overall greater satisfaction with information received (more info is provided via telephone follow-up). Positive comments for telephone follow-up: knowing who to contact, convenience, being reassured	Negative comments for telephone follow-up: preference for face-to-face contact, missing reassurance of clinical exam, feeling isolated/unsettled and problems with organizing telephone appointments
Bohnenkamp et al (2004) [28]	100% of patients agreed that telenursing increased accessibility of care Less expenses due to less frequent pouch changes	15% of telenursing subjects reported that the camera and new technology embarrassed them
Bouchard et al (2019) [33]	N/A	N/A
Williamson et al (2018) [35]	Telephone follow-up was more convenient for patients than hospital follow-up; patients did not have to rely on, or feel they were inconveniencing relatives or friends who would usually take them to hospital appointments, which promoted independence; easier to manage their day Patients found telephone follow-up reassuring and said they found it easier to self-manage than when they were receiving hospital follow-up telephone follow-up provided them with privacy that they perceived was not available at their hospital appointments	N/A
Spoelstra et al (2016) [24]	N/A	N/A
Brett et al (2018) [36]	Phase 1: Women were generally positive about the concept of an app to provide info and support and all of them see great potential in the app for helping women cope with issues that may arise when taking adjuvant endocrine therapy; they also highlighted the accessibility of the app Phase 2: Women reported that the user interface was clear with intuitive controls and user satisfaction was good from the usability tasks Phase 3: Pilot of the app—the participants reported that downloading and navigating the app was straightforward and that it was user friendly	Phase 1 device (eg, phone) required; app needed to be easy to download and simple to navigate.
Steeb et al (2019) [37]	38.5% (72/187) thought that such apps are useful for patients; 42.6% (78/183) voted that skin cancer apps can supplement or support professional skin cancer screening by a physician 59.1% of the patients (110/186) would download a skin cancer app recommended by their physician Men were generally more willing to download an app that has been recommended by their physician than women (P=.02)	76.1% (140/184) figured that skin cancer apps cannot replace skin cancer screening performed by a physician Patients aged >61 years did not think that skin cancer apps can replace the physician in comparison to those under the age of 61 years (P=.02) and would rather read a printed brochure on skin cancer than download an app (P<.001)
Rossen et al (2019) [23]	Training and support in utilizing health technology for rehabilitation	The unreceptive group has a higher representation of vulnerable individuals that are older, with lower educational level, live alone, currently smoke, and with more chronic comorbidities Unreceptive-group experience technology-specific barriers with significantly lower scores in dimensions related to their skills, motivation and user experiences
Rodler et al (2020) [29]	High response rates indicative of rapid acceptance of telehealth services by patients during pandemic despite difficulties of applicability in an aging population with lower email access or with hearing impairment; virtual communication was established quickly directly or through aiding relatives or partners	Patients value personal interactions with their treating physicians greatly; patient–physician distancing can be perceived as a bigger toll than the risk of COVID-19

^a^N/A: not applicable.

### Facilitators

Five studies [27,31,32,34,35] found convenience to be a factor for technology acceptance, making it the most often cited facilitating factor. Four articles [26,28,29,36] mentioned increased accessibility to care as a reason for favoring telemedicine acceptance. Four articles [21,30,36,37] reported that a positive experience of telemedicine increased the likelihood of technology acceptance, and 3 studies [21,30,36] stated the importance of having appropriate technical knowledge and support as a facilitating factor. Other factors included usability [30], lower cost [28], physician recommendation [37], and more privacy [35]. These facilitators are categorized within the theoretical framework of the UTAUT (Table 5).

**Table 5:** Facilitators and barriers within the context of the Unified Theory of Acceptance and Use of Technology model.

	Facilitators	Barriers
Performance expectancy	Increased accessibility Decreased cost Improved privacy	Preference for conventional care Confidentiality concerns
Effort expectancy	Convenience Usability	Technical difficulties
Social influence	Physician recommendation	Negative perceptions of telemedicine
Facilitating conditions	Having appropriate technical know-how and support	None found

### Barriers

Six articles [21,25,26,31,34,37] found that preferences for conventional care along with negative perceptions of telemedicine were barriers to telemedicine acceptance. Participants from 4 articles [23,30,34,36] were concerned about technical difficulties, while those from another 4 articles [25,26,28,31] raised concerns about confidentiality in the adoption of telemedicine. These barriers are categorized within the theoretical framework of the UTAUT model (Table 5).

### Acceptance Across Different Types of Telemedicine

In 2 early studies [25,31] on the acceptance of teleconsultation as a substitute for in-person consults, patients were hesitant to fully transition to teleconsultation.

Participant acceptance of technology in monitoring and follow-up of their oncological condition was explored in 7 studies [20,22,26,27,30,32,38]. Of these, 6 studies [18,20,22,27,32,33] reported a generally favorable attitude toward acceptance of telemedicine; none of these studies went as far as to conclude that telemedicine can be used to completely replace traditional in-person consult for follow-up care.

Acceptance of telemedicine related to psychosocial support and nursing care was studied in 5 studies [21,28,33-35]; 2 articles [28,35] reported a preference for telemedicine. The other 3 articles [21,33,34] reported no difference in preference between telemedicine versus control (traditional face-to-face support).

In both articles using technology as an adjunct for prompting adherence to treatment, participants were found to be generally receptive [24,36].

Three studies [23,29,37] explored participants’ acceptance of technology without specifying the type of telemedicine intervention. One study [23] found that older patients were among those less likely to accept telemedicine as an adjunct to traditional care. Two studies [29,37] reported unfavorable acceptance of telemedicine as a replacement to conventional care. Of particular interest to the ongoing global pandemic was a study [37] on patients’ perspectives on adoption of telemedicine as a response to the COVID-19 pandemic, in which it was noted that the majority of patients (65.9%) were not inclined to continue using telemedicine, because they greatly value personal interactions with their treating physicians. Steeb et al [37] concluded that patient–physician distancing can be perceived as eliciting a bigger toll than the risk of COVID-19.

## Discussion

### Principal Findings

To the best of our knowledge, this is the first scoping review to explore the acceptance of telemedicine among older adults patients with cancer. Our review of 19 primary studies indicated that numerous facilitators and barriers to acceptance of telemedicine exist. Because no study employed a theoretical behavioral framework in their methodology, it was not possible to map facilitators and barriers to UTAUT constructs with great precision. It was possible, however, to classify factors broadly according to the original definition [13] of each construct. We observed that all 4 UTAUT constructs play a role in determining behavioral use (Table 5).

Although facilitators and barriers to telemedicine acceptance in the older adult population were reported in most studies, there was a lack of studies employing methods beyond descriptive methods. None of the studies explored the effectiveness of tailored interventions to address facilitators and barriers to the acceptance of telemedicine by older adult populations; facilitators and barriers would likely differ across different cultural contexts and by type of telemedicine studied—this is a gap in our current knowledge of the use of telemedicine. Studies should aim to illustrate potential facilitators and barriers to telemedicine adoption while exploring the efficacy of different policies to address these factors within local settings. For example, common barriers identified in this review, such as negative perceptions of telemedicine, technical difficulties, and confidentiality concerns, can be easily targeted in education campaigns. The effectiveness of such campaigns can be evaluated and adapted to different local contexts. Policymakers may consider using the UTAUT model as a theoretical basis in designing such interventions.

While most studies showed positive acceptance of telemedicine as an adjunct to traditional care models, there was a paucity of articles that could confidently declare that the telemedicine alternative offered could completely replace the traditional modality. Older adult patients appear to still cherish the opportunity for face-to-face consults, and some studies [25,26,28,29,31,37] reported confidentiality concerns with technology. One article [37] was particularly illuminating—faced with the COVID-19 pandemic, a lot of older adult patients (who are also more vulnerable to complications of COVID-19) were forced to adopt telemedicine as an alternative to in-person consultations, these older adult patients may otherwise not have tried this alternative. Nonetheless, the majority were not inclined to continue adopting such alternatives even if the pandemic continued [37]. This study [37] affords us an interesting view into older adult cancer patients’ psyche in their attitudes toward telemedicine and suggests that more work is needed to examine the facilitators and barriers to telemedicine acceptance among older adults.

Five studies [22,24,28,31,34] reported level of satisfaction among their study population. Although there is generally high satisfaction reported in the use of telemedicine in these studies, acceptance of telemedicine among their study population remains inconclusive. For example, Mair et al [31] found that despite having all study participants reporting satisfaction with telemedicine care, 50% of participants felt telemedicine could not fully replace face-to-face consultations. While participants may be satisfied with the performance of a telemedicine technology, it does not naturally follow that they accept the use of telemedicine over conventional care methods. Acceptance of a telemedicine alternative and preference for its use over traditional care models are, therefore, different concepts from satisfaction. For telemedicine alternatives to enjoy widespread sustained adoption by the older adult patients when implemented by health care systems and organizations, future studies could examine the relationship between satisfaction and acceptance of telemedicine and analyze factors affecting the correlation between the two constructs.

The findings of our review may have implications for health care and public health policymakers in the context of telemedicine implementation. Caring for an older adult with cancer is complex. Health care innovation requires corresponding changes in behavioral patterns among the older adult population to realize its full potential. While some traditional barriers to health care access may be overcome, the acceptance of telemedicine itself is a behavior change, challenging the accepted health care delivery norm. In the adoption of telemedicine to aid in health care, the older adult population faces a whole new distinct set of facilitators and barriers, and an understanding of these is important to increase telemedicine acceptance [39]. As shown in our review, this may be done with the help of theoretical frameworks such as the UTAUT.

With the ongoing COVID-19 pandemic, which appears to be accelerating the worldwide adoption of telemedicine, it is inevitable that many patients will face the prospect of having to integrate telemedicine into their care routines—within the 6-month period from January to June 2020, there were 543 articles published on telemedicine-related literature [40]. New facilitators or barriers might emerge from this pandemic-induced adoption of telemedicine. Understanding these complex factors through the lens of a theoretical framework could provide a solid foundation for policymakers to navigate these challenging times. Moving forward, it may also be worthwhile for public health researchers and professionals to adopt a mixed methods approach to understand how existing theories of technology acceptance may be extended to account for COVID-related factors. For example, the use of inductive grounded-theory methodologies may help to uncover novel themes and constructs not in existing theoretical frameworks.

### Limitations

We acknowledge several limitations in our review. First, while we took the definition of older adults to be 65 years and older, it was not practical to identify only articles that contain a sample population of this age range. Doing so would have severely limited the number of articles available for analysis and would have excluded numerous studies in which the majority of the sample population were 65 years or older. We would have missed out on potentially available evidence in the literature pertaining to older adult patients and telemedicine. We, therefore, also included any studies in which a subgroup analysis on participants 65 years and older was performed. This compromise meant that a proportion (albeit, less than half) of the sampled population among included articles was younger than 65 years of age.

Second, the definition of telemedicine is very wide, and a range of telemedicine interventions had to be considered. This contributed to the heterogeneity of the studies included in this review.

Third, all the studies were conducted in Western high-income nations. This limits the generalizability of our findings. Attitudes of older adults with cancer toward telemedicine may be influenced by cultural factors and education level. The mean education levels of low- or middle-income nations could also be different from those of the sample populations included in this review; thus, the adoption of telemedicine in low- or middle-income nations by older adults with cancer might encounter additional difficulties.

Fourth, telemedicine is a rapidly emerging field, and its emergence has been expedited by the COVID-19 pandemic. While we were able to include articles from database inception to September 2020, it is likely that more studies have been published since September 2020.

### Conclusion

The findings of our scoping review have important implications for future research. In a world grappling with the COVID-19 pandemic, telemedicine offers an alternative model of care and is here to stay. Our review has identified research gaps to be addressed. Future studies are necessary to understand the facilitators and barriers to telemedicine acceptance in older adults with cancer, with a view to investigating interventions to address barriers.

## Appendix

Multimedia Appendix 1 Supplementary tables.

## Abbreviations

PICOS: population, intervention, comparison, outcomes, study design
UTAUT: Unified Theory of Acceptance and Use of Technology
