# Supplementary material for: Telemedicine Acceptance Among Older Adult Patients With Cancer: Scoping Review
Source: J Med Internet Res. 2022 Mar 29;24(3):e28724. doi: 10.2196/28724 (PMC9006130; doi:10.2196/28724)
Supplement: Multimedia Appendix 1 [file jmir_v24i3e28724_app1.docx]

*Supplementary Table 1*. PICOS framework.

| Population (P) | Older adults who are cancer patients (i.e., ≥ 65 years of age) |
| --- | --- |
| Intervention (I) | Telemedicine initiative/intervention |
| Comparison (C) | Standard care (where appropriate, in studies with comparative designs; e.g., hospital or face-to-face cancer care) |
| Outcomes (O) | Acceptability and/or satisfaction towards telemedicine |
| Study design (S) | Quantitative, qualitative, or mixed-methods designs |

*Supplementary Table 2*. Search strategy.

| **Database** | **Strategy** |
| --- | --- |
| **Pubmed** | ("telemedicine"[MeSH Terms] OR "telemedicine"[All Fields] OR "telemedicine s"[All Fields]) AND ("neoplasm s"[All Fields] OR "neoplasms"[MeSH Terms] OR "neoplasms"[All Fields] OR "neoplasm"[All Fields]) AND ("attitude to health"[MeSH Terms] OR ("attitude"[All Fields] AND "health"[All Fields]) OR "attitude to health"[All Fields]) |
| **Embase** | ('telehealth'/exp OR 'telehealth') AND ('neoplasm'/exp OR 'neoplasm') AND ('patient attitude'/exp OR 'patient attitude') |
| **PsycInfo** | exp telemedicine/ and (exp neoplasms/) and (exp client attitude/ or exp client participation/ or exp treatment barriers/ or exp treatment compliance/) |
| **CINAHL** | (MH "Telehealth+") AND (MH "Neoplasms+") AND (MH "Attitude to Health+" OR MH "Health behaviour+") |
| **COCHRANE** | [mh Telemedicine] AND ([mh Neoplasms])AND [mh "Attitude to health"] |
